# Clozapine-Induced Granulomatous Interstitial Nephritis: A Case Report

**DOI:** 10.7759/cureus.46971

**Published:** 2023-10-13

**Authors:** Nismat Javed, Anandu M Anto, Rabih Nasr, Kalpana A Uday

**Affiliations:** 1 Internal Medicine, BronxCare Health System, Bronx, USA; 2 Nephrology, BronxCare Health System, Bronx, USA; 3 Internal Medicine, Icahn School of Medicine at Mount Sinai, New York, USA; 4 Nephrology, Grand Concourse Dialysis Unit, Bronx, USA

**Keywords:** clinical presentation, prognosis, management, granulomatous interstitial nephritis, clozapine

## Abstract

Granulomatous interstitial nephritis is a rare form of tubulointerstitial nephritis and has been uncommonly observed with clozapine usage. Additionally, the progression of the disease to manifest as renal failure requiring dialysis is also uncommon. We describe a case of a 56-year-old female who presented with syncope and was diagnosed with granulomatous interstitial nephritis on biopsy. While hemodialysis may play a role in the management of the disease, steroids provide a definitive treatment. Large-scale studies are needed to evaluate the role of clozapine in causing interstitial nephritis and the characteristics of these features to establish a therapeutic goal.

## Introduction

Granulomatous interstitial nephritis is a relatively rare condition detected in about 0.5 to 0.9% of all renal biopsies and accounts for 6% of patients diagnosed with tubulointerstitial nephritis [1,2]. Clozapine-induced granulomatous interstitial nephritis is a relatively rare phenomenon that has not been extensively reported in the literature [3]. We present a case of a 56-year-old female who presented with syncope and worsening renal function. She was diagnosed with granulomatous interstitial nephritis on biopsy and treated with prednisone.

## Case presentation

A 56-year-old female was brought in by Emergency Medical Services after a syncopal episode. The episode was acute in onset, followed by loss of consciousness for a few seconds. She had been having these episodes for the past one month. These episodes started after changes were made to her clozapine dose about three months ago. She was previously taking 100 mg of olanzapine in the morning and 400 mg at night, but the dose was increased to 500 mg during the night before the onset of the symptoms. Her past medical history was significant for hypertension, chronic kidney disease, asthma, deep venous thrombosis, sciatica, osteoarthrosis, post-menopausal atrophic vaginitis, recurrent urinary tract infections, schizophrenia, and chronic tremor. Social and family history did not reveal any significant information.

**Figure 1:**
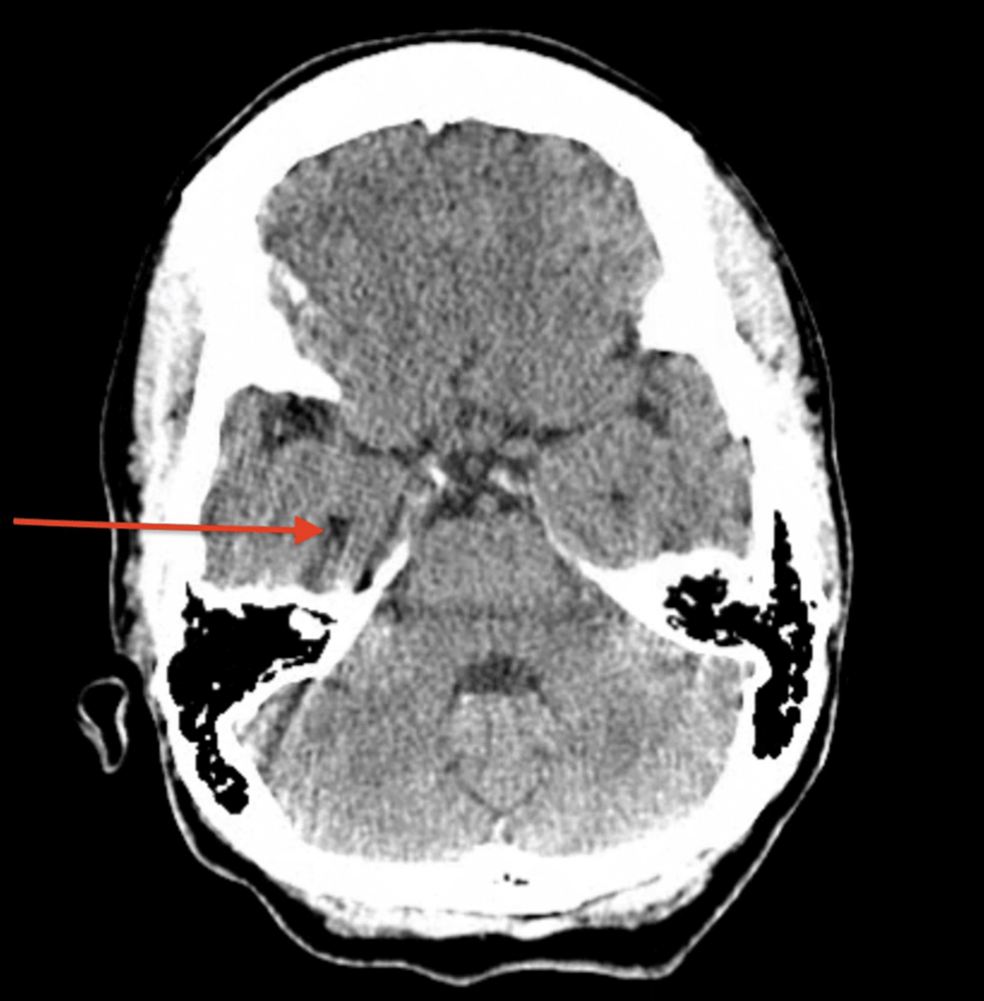
CT scan (coronal view) of the head without contrast The image is showing no acute abnormality (as demonstrated by the red arrow).

On presentation, vital signs revealed a temperature of 98.8 F, blood pressure of 108/67 mmHg, pulse of 98/minute, and oxygen saturation of 96% on room air. There were no orthostatic changes in the vital signs. Physical examination revealed left shoulder tenderness and power was grossly normal. A cardiovascular examination revealed normal heart sounds. The abdomen was soft and non-distended with normal bowel sounds. Laboratory investigations revealed anemia (hemoglobin:10.5 g/dl (reference range: 12-16 g/dl)), hyperkalemia (potassium: 5.1 mEq/L (reference range: 3.5-5.0 mEq/L)), elevated creatinine (1.8 mg/dl (reference range: 0.5-1.5 mg/dl)) and elevated d-dimer (1256 ng/mL (reference range: 0-230 ng/mL)). Urinalysis revealed leukocyte esterase, pro b-type natriuretic peptide (proBNP) was elevated, and mild protein (30 mg/dl). CT brain did not reveal any significant abnormality (Figure 1). 

**Figure 2:**
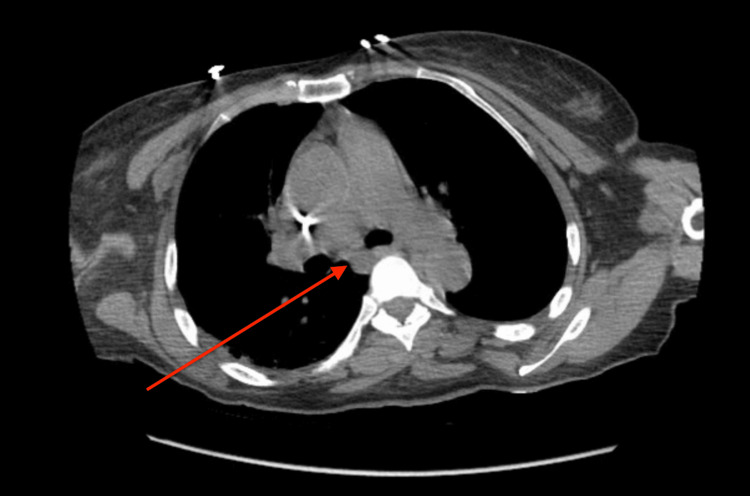
CT scan of the chest without contrast The image is showing no hilar lymphadenopathy (as demonstrated by the red arrow).

Ultrasound of bilateral lower extremities did not reveal deep venous thrombosis. VQ scan (ventilation/perfusion scan of lungs) revealed a low probability of pulmonary embolism. CT scan of the chest did not reveal any evidence of abnormality (Figure 2).

An echocardiogram revealed a preserved ejection fraction and a moderately dilated left atrium. Levels of clozapine were significantly elevated hence it was held. Glomerular serologies revealed positive anti-nuclear antibodies in low titers. Complement levels were normal. Antibodies to double-stranded DNA were not elevated. Ultrasound of the kidneys showed renal cysts and medical renal disease. Fractional excretion of sodium was significant for intrinsic renal disease.

**Figure 3:**
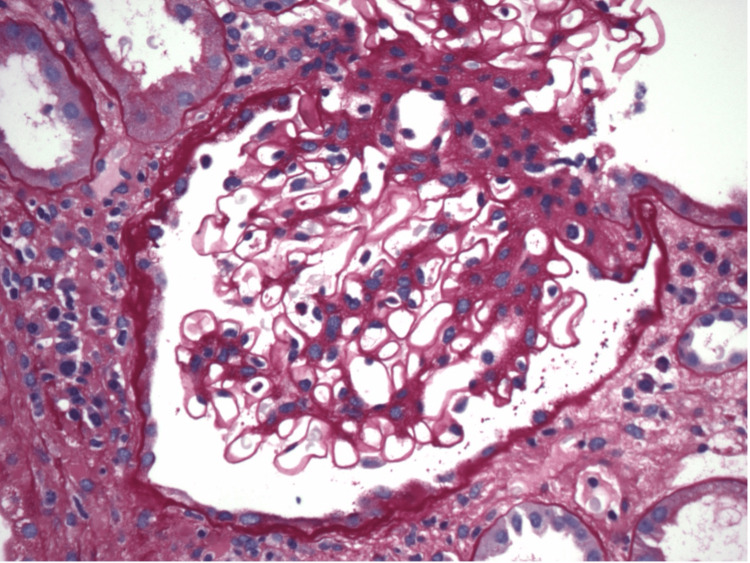
Non-sclerotic glomerulus with mesangial sclerosis and patent capillary lumina

**Figure 4:**
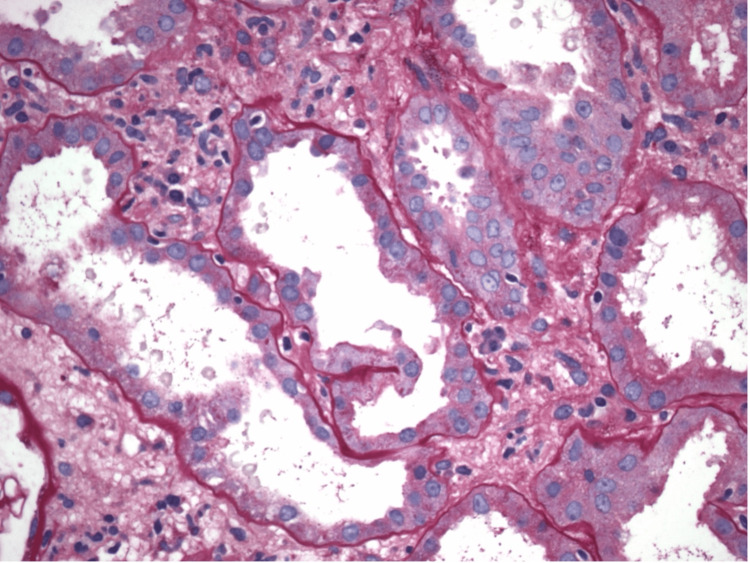
Proximal tubule showing acute tubular injury

**Figure 5:**
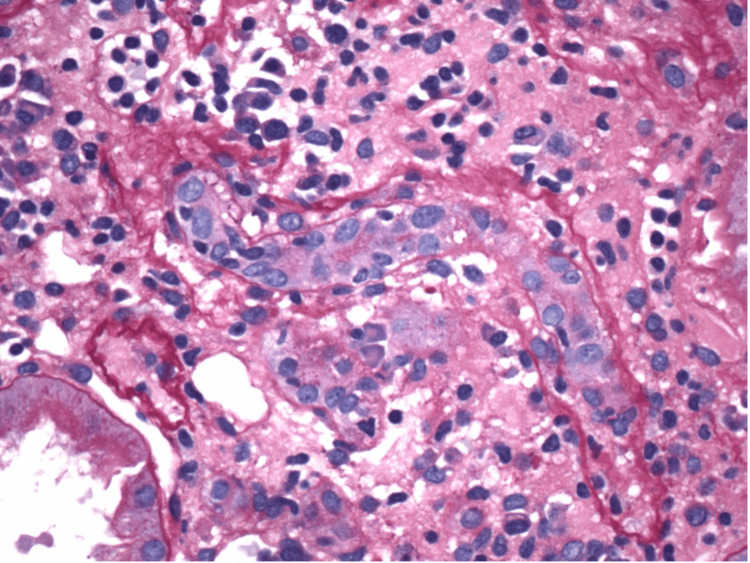
Tubulitis with edema evident

**Figure 6:**
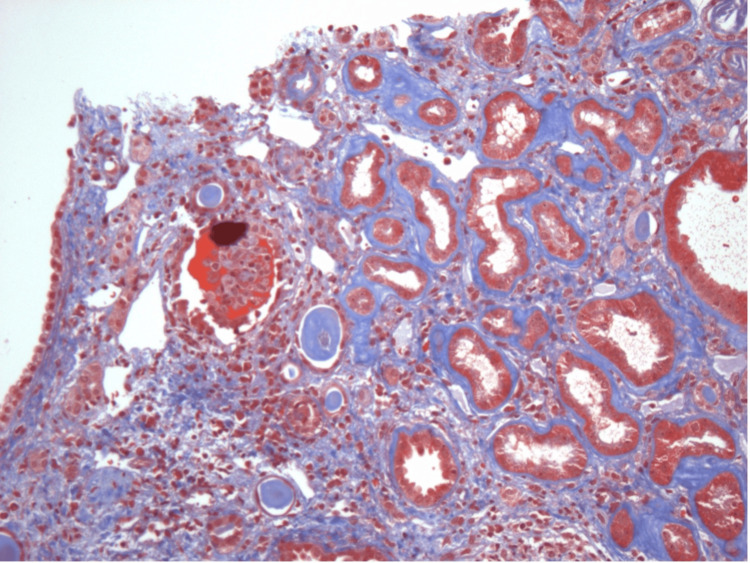
Interstitial fibrosis with mononuclear cells and a few eosinophils

**Figure 7:**
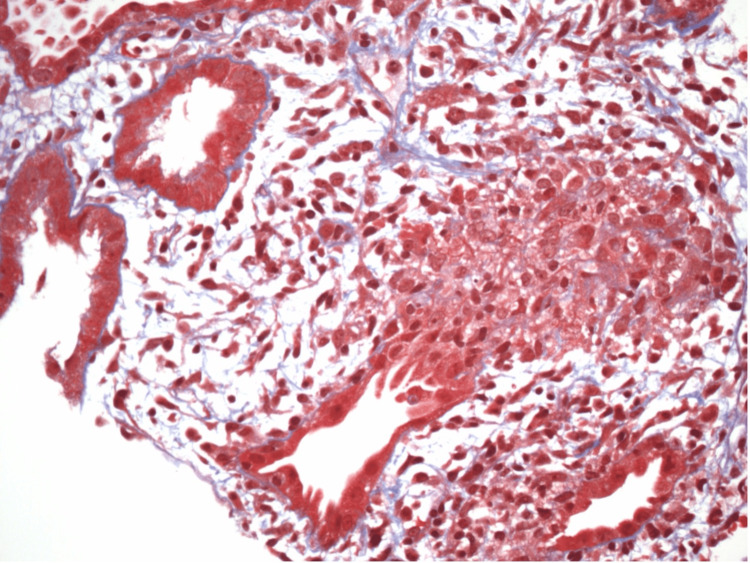
Non-necrotizing granuloma visualized

Her renal function continued to deteriorate until day 6 of hospitalization. On day 10 of hospitalization, she started experiencing myoclonic jerks which were attributed to uremic encephalopathy and the patient was started on hemodialysis. She received four sessions of hemodialysis. A renal biopsy was performed. Pathology revealed granulomatous interstitial nephritis (Figures 3-7).

There was no evidence of immune complex-mediated lupus nephritis on the kidney biopsy. Subsequently, we obtained angiotensin-converting enzyme levels which were high. A pulmonary consult was requested and suggested that there was a low suspicion of sarcoidosis, hence, bronchoscopy was not performed. Additionally, the patient did not report any dry eyes or mouth and Sjogren antibodies were negative. There were no monoclonal proteins in urine immunofixation. The serum kappa to lambda light chain ratio was normal. The 24-hour urine collection showed 192.6 mg of protein and 31 mg of albumin in a volume of 900 mL. These findings were consistent with tubular proteinuria as seen in interstitial nephritis.

The patient was started on prednisone 70 mg once daily on day 19 of hospitalization and clozapine was also discontinued on day 19. She was switched to haloperidol 5 mg every 12 hours and depakote 500 mg every 12 hours with plans for outpatient follow-up with psychiatry on day 23 of hospitalization. Her hospital course was also complicated by new onset atrial fibrillation for which apixaban 2.5 mg every 12 hours, and metoprolol 12.5 mg every 12 hours were started on day 7 of hospitalization. Her renal function began to improve on day 20 of hospitalization, and she was started on a prednisone taper to be continued in the outpatient setting. Therefore, temporary vascular access was removed as creatinine downtrended on day 25 of hospitalization, and she did not require any further dialysis. She was discharged to a short-term rehabilitation facility with an appointment to the renal clinic after 28 days of hospitalization.

## Discussion

Clozapine is an antipsychotic medication that is used in the treatment of schizophrenia and other psychiatric disorders, for example, bipolar disorder [4,5]. Clozapine-induced granulomatous interstitial nephritis is relatively uncommon. The median age of the patients varies from 25 to 40 years of age [4,5]. The disease is more common in women as compared to men [3,6]. A few cases have also been reported in older individuals and in male patients [7]. Although no specific risk factors have been identified, patients with a history of schizophrenia and resistant mood disorders are more likely to develop the disease [7]. The majority of the patients developed nephritis within two weeks [6]. There is no conclusive link between the dosage of medication and the rate of development of interstitial nephritis [6].

Postulated mechanisms for the injury include a re-introduction to the inciting agent or an increase in the dose of the inciting agent [1]. Additionally, a potential immune response to the antigenic drug can also manifest as a similar kidney injury [1]. The drug can also bind to the tubular basement membrane acting as a hapten leading to cell-mediated reaction [1].

Syncope, defined as a transient loss of consciousness secondary to orthostasis or other causes, is an uncommon manifestation of granulomatous interstitial nephritis. Clozapine has been observed to cause autonomic dysfunction that can manifest as orthostasis, tachycardia, and neuropathy [7]. Most patients present with respiratory symptoms or an asymptomatic elevation in serum creatinine [6,8]. High levels of clozapine could have contributed to the symptoms of our patient.

Laboratory investigations were generally found to be non-specific. Inflammatory markers were raised in most of the cases, contrary to our patient [9,10]. In our case, the patient additionally had anemia and hyperkalemia that had not been observed previously [9,10]. Mild proteinuria was also observed in our case. Elevation of serum creatinine has been observed in the majority of the cases [7-11].

About 40% of the cases with interstitial nephritis secondary to clozapine use require dialysis [6,12]. Usually, the kidney injury resolves with discontinuation of the drug and may need hemodialysis briefly [6]. Prednisone has also been studied as a mainstay of treatment with prednisone taper providing an adequate response in most of the patients [3]. In rare cases, the kidney function might not completely normalize [6]. In patients with disease refractory to steroids, mycophenolate mofetil is one of the therapeutic options that is well tolerated by patients in studies [13]. Treatment-resistant patients are more likely to have a recurrence of the same condition [6]. However, prognosis in most cases is favourable [7-11].

## Conclusions

Clozapine-induced granulomatous interstitial nephritis is a detrimental condition that can result from an immune-mediated reaction as a result of increased dosage or at the onset of medication administration. While dialysis plays a crucial role in treatment, the extent of the damage can be reduced and reversed through early identification and subsequent discontinuation of the offending agent. Management, therefore, requires both source control and appropriate initiation of dialysis. Furthermore, steroids also have a beneficial role to play in this regard in terms of source control. There is a need to investigate the disease further in large-scale studies.
